# Impact of Diagnostic Practices on the Self-Reported Health of Mothers of Recently Diagnosed Children with ASD

**DOI:** 10.3390/ijerph13090888

**Published:** 2016-09-07

**Authors:** Phil Reed, Lucy Picton, Nicole Grainger, Lisa A. Osborne

**Affiliations:** 1 Department of Psychology, Swansea University, Singleton Park, Swansea SA2 8PP, UK; 523722@swansea.ac.uk (L.P.); 633683@swansea.ac.uk (N.G.); 2 Abertawe Bro Morgannwg University Health Board, Swansea SA2 8QA, UK; lisaanneosborne@yahoo.com

**Keywords:** ASD diagnosis, parent health, anxiety, diagnostic coherence, ASD

## Abstract

*Objectives*: Obtaining a diagnosis of an Autism Spectrum Disorder (ASD) for a child is a pivotal point in developing the treatment plan for the child but can also be regarded as highly stressful by parents. The current study examined the impact of different aspects of the diagnosis process on the self-reported mental health of mothers of children undergoing a diagnosis for ASD in a cross-sectional cohort design. *Methods*: One-hundred-fifty-eight mothers of consequently diagnosed children with ASD participated. The severity of the children’s ASD and their intellectual functioning was assessed within twelve months of the diagnosis, and the mothers completed a psychometric assessment battery including the Hospital Anxiety and Depression Scale, General Health Questionnaire, and Questionnaire on Resources and Stress. *Results*: The actual time from first reporting a problem to obtaining a diagnosis, and the speed of the diagnostic process from first to last appointment, were both negatively related to patenting stress. In contrast, mothers’ perceptions of the speed and helpfulness of the process were negatively related to levels of anxiety and depression. The number of professionals involved in the process and the perceived coherence of the diagnosis were also negatively related to aspects of mothers’ functioning. *Conclusions*: Care is needed to help mothers through the diagnostic process with regard to their own functioning. Providing information and help sources throughout the process, while keeping the number of professionals involved to a minimum, may improve the parent perception of the process and reduce the negative impacts of the diagnosis on the family as a whole.

## 1. Introduction

Obtaining a diagnosis of Autism Spectrum Disorder (ASD) is a pivotal point in developing a treatment plan for the child [1]. The diagnosis allows parents to access a range of services [2,3], and develop their relationship with their child [4,5,6,7,8]. Although offering some clear benefits, the process of obtaining a diagnosis of ASD for a child is often regarded as highly stressful by parents [9,10,11,12,13,14]. It is known that parental stress can impact negatively their ability to parent their child [15,16,17], leading to more acute child behaviour problems [17,18], and impeding child progress on many psycho-social interventions [19,20,21,22]. However, it is not known which aspects of the diagnostic process are most strongly related to which aspects of parental functioning, and, hence, it is difficult to suggest exactly how to improve diagnostic services to benefit children and parents.

The limited research to date suggests that although parental reactions to diagnosis vary greatly [13,23,24], barely 50% of parents are satisfied with the diagnostic process [3,6]. A common theme emerging from studies of parental reactions to the diagnostic process is that this process is poorly organised or structured and lacks coherence, and that this reaction appears to occur across different diagnostic systems in different countries [10,12,13,14,19,20,24]. Parental satisfaction with the diagnostic process increases with speed of obtaining a diagnosis [25], the clarity of the diagnosis received [21], and the perceived quality of the information provided [26,27]. Moreover, the nature of communication with professionals during diagnosis predicts parental satisfaction—the fewer professionals that parents need to see in order to obtain a diagnosis [12], and the greater the perceived professionalism of the person giving the diagnosis [9,14,28], the greater the parental satisfaction. However, although these studies have identified aspects of the diagnostic process that are related to parent satisfaction, they have not identified whether they also impact parent functioning. This is an important distinction, as it is not clear that satisfaction with the diagnostic predicts positive parental functioning. Indeed, there are suggestions that too speedy a diagnostic process may result in worse psychological functioning for the parent as a result of them having less time to adjust their lives and coping strategies to this label [29]. 

Parents of children with ASD display a range of areas of impaired functioning, any of which might be exacerbated by aspects of the diagnostic process. There are very high levels of parenting stress for this population [30,31] associated with impaired immune function responses [32], and these levels of stress reach a peak during the diagnosis process [11]. In addition, parents of children with ASD report relatively high levels of mental health problems, such as anxiety and depression [33,34], and report poor health-related quality of life (HR-QoL) [35,36]. In addition to the negative impacts of high levels of psychological distress and ill health on parenting ability [17] and child development [14,18,22,29], it can also impact negatively on parental ability to communicate essential information to professionals, or their ability to comprehend and integrate medical information [37]. Highly stressed or ill parents may skip doctors’ appointment, stall therapies, or avoid visiting children in residential facilities [38], and may suppress discussions about the child’s diagnosis.

Thus, it has been consistently acknowledged that parents play a pivotal role in the prospects for the child with ASD [18,29,39,40], and that those parents suffer from poor health and psychological well-being [30,35], which has, in turn, a negative impact on the prospective outcomes for the child with ASD [22,29]. It is also commonly suggested that many of the early parental stressors revolve around the diagnostic process [14]. However, relatively little is known about the precise aspects of the diagnostic process that impact on such functioning. This information is critical in developing a better understanding of how the diagnostic process could be improved. Given this, the main aims of the current study were to survey the diagnostic experiences and psychological functioning of mothers of recently ASD-diagnosed children, and to relate these diagnostic experiences to a range of functioning domains including: anxiety, depression, HR-QoL, and parenting stress. Mothers were the focus of this exploratory study as there is some evidence that fathers and mothers may react differently to the stresses of parenting a child with ASD, and we believed that we would be more likely to successfully recruit a sufficiently large sample of mothers but not fathers for analyses.

## 2. Method

### 2.1. Participants

The mothers of 175 consecutive children who had been diagnosed with ASD within the last 12 months were approached and asked if they would be willing to participate in research about their feelings about the diagnostic process. Of these mothers, 158 (90%) agreed to participate. The mean age of the participating mothers was 39.34 (+8.24, range 24–59) years; 99 (63%) were married or civil partnership, 28 (18%) were in another form of relationship, and 31 (19%) were single, divorced, or widowed. In terms of occupation, 18 (12%) were classed as labourers, 15 (10%) as skilled, 49 (31%) as managerial/professional, and 76 (43%) as unemployed or home-makers. The ethnic background of the mothers was: 115 (72%) White, 8 (6%) Mixed/Multiple, 17 (11%) Asian; 14 (9%) Black/African/Caribbean, and 4 (2%) Other.

The children were all diagnosed by consultant paediatricians who were independent from this study, and who all used a combination of the Diagnostic and Statistical Manual (DSM-IV-TR) criteria, the Autism Diagnostic Observation Schedule (ADOS), and clinical judgment, to make the diagnosis. There were 123 (78%) male and 35 female (22%) children, who had a mean age of 8.34 (+4.53, range = 2–18) years: 52 (33%) preschool age (2–5 years); 66 (42%) primary school age (6–11), 31 (20%) secondary school age (12–16); and 9 (5%) post-secondary age. Of the children, 93 (59%) received a diagnosis of autism, 24 (15%) a diagnosis of Asperger syndrome, and 41 (26%) pervasive developmental disorder not otherwise specified. The severity of the ASD was assessed using the standardised severity score [41] of the Autism Diagnostic Observation Schedule [42], and the mean was 6.55 (+1.81; range = 3–10). The mean speed of the diagnostic process experienced (from first appointment to receiving a diagnosis according to the medical records) was 16.75 (+11.15; range = 1–49) months.

The children’s functioning was independently tested using the ADOS (using Calibrated Severity Score to get a measure of autism severity), the Leiter International Performance scale (using the overall standard score to get a measure of nonverbal intellectual functioning), and the British Picture Vocabulary Scale (using the overall standard score to get a measure of verbal functioning). The characteristics of the sample are shown in Table 1.

Ethical approval for this research was given by the Department of Psychology’s Ethics Committee at a UK University.

### 2.2. Materials

The Autism Diagnostic Observation Schedule (ADOS) [42] is a semi-structured standardized observation that measures autism symptoms in social relatedness, communication, play, and repetitive behaviours. A standardised severity score (CSS) can be calculated to assess the severity of symptoms across modules [41]. The CSS has a range of 1–10, with 4 being the cut off for ASD, and 6 for autism.

Leiter International Performance Scale-Revised (Leiter-R) [43] is a measure of intellectual abilities of individuals (aged 2 to 20 years) with significant communication disorders, cognitive delays, and various types of learning disabilities. It has been used extensively for the assessment of children with ASD, and provides a total nonverbal IQ score (mean = 100 and SD = 15). The reliability for the full IQ for different age groups varies from 0.91 to 0.93, and the test–retest reliability varies from 0.61 to 0.90. It has a 0.85 correlation with the Wechsler Intelligence Scale for Children (WISC) full scale IQ measure.

The British Picture Vocabulary Scale (BPVS) [44] is derived from the Peabody Picture Vocabulary Scale, and measures receptive language ability. The BPVS is standardized for use on children in the UK between 3 and 17 years old, and gives an age equivalent score for this ability. It has an internal reliability of 0.93, and has a 0.59 correlation with the Reynell Comprehension Scale.

The Hospital Anxiety and Depression Scale (HADS) [45] assesses levels of anxiety and depression. It has since been widely employed in primary care settings, and contains 14 question items (7 for anxiety and 7 for depression), that relate to the last week, and takes about 10 min to complete. The test-retest reliability varies for a population of parents with ASD of 0.86 for anxiety and 0.74 for depression.

The General Health Questionnaire (GHQ-28) measures a range of psychiatric and health problems, and is divided into 4 sub-scales: somatic symptoms, anxiety and insomnia, social dysfunction, and severe depression. The internal reliability of the overall scale is above 0.90. 

The Questionnaire on Resources and Stress (QRS-F) [46] is a 52-item, self-administered, true/false tool, designed to measure parental perceptions of the impact of a developmentally delayed, or chronically ill, child on other family members. It takes about 10 min to complete. It consists of four sub-scales (Parent and Family Problems, Pessimism, Child Characteristics, and Physical Incapacity). These scores summate to produce a Total Stress Score. The internal reliability of the total stress scale is 0.89, with the sub-scales ranges from 0.77 to 0.85. 

Diagnostic Experience Scale (DES) to evaluate the mothers’ perceptions of the diagnostic process was developed for this study. The mothers were asked to rate their experience of the diagnostic process on a five-point scale (1–5), where 1 = very poor and 5 = very good, in terms of its: speed, the inter-personal skills of the professionals, the communication ability of the professionals, helpfulness in understanding ASD, and coherence. These questions were chosen on the basis of the aspects of the process that parents had mentioned in many qualitative studies of their experiences of getting a diagnosis of ASD for their child (see Introduction). The internal reliability (Cronbach α) of the scale for this sample was calculated at 0.78, with no item producing an increase in this value if it was removed.

### 2.3. Procedure

Following the diagnosis being confirmed, the mothers were approached and asked if they would like to participate in the study. Once consent had been given, a psychologist visited the mothers and tested the child with the ADOS, Leiter, and BPVS scales. The mothers also completed the HADS, GHQ-28, QRS-F, and DES questionnaires at the same time. The mothers were also asked their child’s age, and the age at which they first noticed a problem with their child. In addition, permission was sought from the mother to access the medical records of their child, and these were explored to ascertain the age time taken for the diagnosis process (from first appointment to receiving a diagnosis), the child’s age at receiving a diagnosis, and how many professionals were involved in the process.

## 3. Results

Table 2 shows the mean values for the mothers’ ratings of the diagnostic process (top row), and the relationship between these values and mothers’ age, the child variables (age, autism, intellectual and verbal functioning), and the diagnostic variables (child age at diagnosis, age when first noticing a problem, time to diagnosis, duration of the diagnosis period, and the number of professional involved). Inspection of these data reveals that the mothers’ age did not impact on their views of the diagnostic process. There were very few child characteristics that impacted on the mother’s view of the process; the only variable that had any strong correlations with these views was child verbal functioning, which was positively associated with both the perceived speed of the process and communication with professionals. There were few reliable correlations between the objective facts about the diagnostic process and the mothers’ views about the process. The only two reliable associations being negative ones between the child’s age at diagnosis and the perceived speed of the process (the older the child at diagnosis the worse was the perceived speed), and the number of professionals involved and the coherence of the process (the more professionals the less coherent the process appeared to the mother). However, none of the associations reported in Table 2 were particularly large, and, taking a “rule of thumb” value that an association that accommodates less than 10% variance between variables (i.e., *r* = 0.30) should not regarded as particularly important or useful, the current results suggest these background variables do not strongly associated with views of the diagnostic process.

The relationship between the mothers’ functioning variables (anxiety, depression, HR-QoL, and parenting stress) and their age, child characteristics, and the objective characteristics of the diagnostic process, are shown in Table 3. Backward stepwise multiple regressions was conducted for each of the four parent functioning variables including all of the predictors shown in Table 3 (and employing a Bonferroni correction to accommodate the multiple testing, *p* = 0.05/4 = 0.0125). The regression conducted for anxiety (HADS-A) revealed a three variable model was statistically significant, F (3, 141) = 11.74, *p* < 0.001, *R*^2^ = 0.200; and had child age at diagnosis (β = 0.188, *p* = 0.088), the time to diagnosis (β = −0.023, *p* = 0.064), and the number of professionals involved (β = 0.714, *p* = 0.001) as predictors. The number of professionals involved was the only independently significant predictor. The regression conducted on depression (HADS-D) revealed no significant model. The regression conducted on HR-QoL (GHQ) revealed a four variable model was statistically significant, F (4, 140) = 6.64, *p* < 0.001, *R*^2^ = 0.159; and had parent age (β = −0.443, *p* = 0.068), child age (β = −0.986, *p* = 0.024), child age at first noticing a problem (β = 1.424, *p* = 0.016), and the number of professionals involved (β = −0.1.164, *p* = 0.063) as predictors. The child age at first noticing a problem was the only independently significant predictor. The regression conducted for parenting stress (QRS) revealed a three variable model was statistically significant, F (3, 141) = 14.06, *p* < 0.001, *R*^2^ = 0.230; and had child verbal functioning (β = −0.166, *p* = 0.001), the time to diagnosis (β = −0.045, *p* = 0.001), and the length of the process (β = −0.128, *p* = 0.005) as independently significant predictors.

Table 4 shows the correlations between the mothers’ ratings of the diagnostic process and their functioning. Backward stepwise multiple regressions were conducted for each of the four parent functioning variables including all of the individual DES predictors shown in Table 4 (and employing a Bonferroni correction to accommodate the multiple testing, *p* = 0.05/4 = 0.0125). The regression conducted for anxiety (HADS-A) revealed a three variable model was statistically significant, F (3, 141) = 24.51, *p* < 0.001, *R*^2^ = 0.343; and had perceived speed of diagnosis (β = −0.338, *p* = 0.013), perceived helpfulness (β = −0.490, *p* = 0.003), and perceived coherence of the process (β = −0.619, *p* = 0.001) as independently significant predictors. The regression conducted on depression (HADS-D) revealed a three variable model was statistically significant, F (3, 141) = 5.64, *p* < 0.001, *R*^2^ = 0.107; and had child intellectual functioning (β = 0.028, *p* = 0.080), perceived speed of diagnosis (β = −0.340, *p* = 0.010), perceived helpfulness (β = −0.318, *p* = 0.016). The perceived speed of the process and its perceived helpfulness were independently significant predictors. The regression conducted on HR-QoL (GHQ) revealed a four variable model was statistically significant, F (4, 140) = 8.31, *p* < 0.001, *R*^2^ = 0.192; and had parent age (β = −0.434, *p* = 0.059), child age (β = −0.734, *p* = 0.067), perceived interpersonal skills of the professional (β = −2.038, *p* = 0.010), and the perceived helpfulness of the process (β = −1.832, *p* = 0.016) as predictors. The perceived helpfulness and interpersonal skills of the professional were the only independently significant predictors. The regression conducted for parenting stress (QRS) revealed a three variable model was statistically significant, F (3, 141) = 11.08, *p* < 0.001, *R*^2^ = 0.191; and had child verbal functioning (β = −0.175, *p* = 0.001), perceived interpersonal skills of the professional (β = −0.819, *p* = 0.049), and the perceived coherence of the process (β = −1.045, *p* = 0.008) as predictors. 

## 4. Discussion

The current report examined relationships between the diagnostic process, both objective and perceived, and several aspects of functioning of mothers of children with ASD. Several objective aspects of the diagnostic process were correlated with mothers’ functioning, most notably with parenting stress (QRS-F), where the shorter the wait to receive the diagnosis, and the quicker the diagnostic process, the greater were the levels of parenting stress. Although these findings appear strikingly counter-intuitive, they extend and replicate those of an earlier study [47] in which a similar relationship between the speed of diagnosis and parenting stress was noted. This previous study [47], however, relied entirely on parental reports of the diagnosis process, and was conducted on a sample that had a very wide range of times since the diagnosis. The current sample was recently diagnosed, as well as the relationships between aspects of the diagnosis process and mothers’ functioning held when inspecting the objective medical records relating to this diagnostic process. These findings suggest that, while an early diagnosis might lead to quicker access to services and beneficial earlier treatment for the child [2], it may also leave mothers unable to develop coping mechanisms for living with this diagnosis [14]. The resultant enhanced level of parenting stress may, in turn, have deleterious consequences for their child’s development [15,16,17], perhaps undoing the benefits of any early treatment [22,29].

Although the objective aspects of the diagnostic process impacted most strongly on parenting stress, mothers’ subjective perceptions of the diagnostic process impacted on a range of functioning variables, most heavily on levels of anxiety (HADS-A). In this regard, perceived speed, helpfulness, and coherence of the diagnosis process were strongly associated with anxiety. The perceived helpfulness of the process also impacted HR-QoL; coherence impacted parenting stress; and the perceived diagnostic speed also was related to depression. While the perceived helpfulness and coherence of the diagnostic process might be expected to impact mothers’ functioning in the period after the diagnosis [10,11,12], the relationship between perceived diagnostic speed with both anxiety and depression, which has been noted previously [25], poses a dilemma for professionals. Parental distress is reduced by having a diagnosis that the parents perceive as speedy [14,25]; however, the objective length of the process has been noted to increase their parenting stress [47]. This finding suggests that care is needed to help the parent through the diagnostic process with regard to their own functioning as well as the child’s, as the former will ultimately impact the latter [18]. The current work, and a number of previous reports based on parents views of the diagnosis process, suggest that providing information and help sources throughout the process [26,27], while keeping the number of professionals involved to a minimum [12,14], may improve the parental perception of the process and reduce the negative impacts of the diagnosis on the family as a whole.

Of course, the current study employed a cross-sectional design, and care must be taken not to interpret the associations as proving a causal relationship between the diagnostic process and parental functioning. For example, it may be that initially more anxious mothers are more dissatisfied with the diagnostic process, or that some third factor causes both anxiety and a lack of satisfaction with the events connected to the diagnosis. Although it would be impossible to set up an experiment to examine the causal relationship between these events, further work could adopt a longitudinal design to see the temporal relationship between these measures. However, even this would be somewhat difficult, as it would necessitate taking anxiety measures, etc., prior to the development of child problems.

The usual caveats should also be given about the representativeness of a sample of mothers who volunteered for such research. In addition, it is not known if these findings would be replicated if a sample of fathers were to be employed. It might also be noted that the age range of the children was large, and it is unclear if these relationships would hold for samples with narrower age ranges. Clearly, exploration of this issue would need a much larger sample size than that employed in the current study, and it might be noted that child age did not seem to correlate with any of the outcome variables strongly. It might be noted that the majority of mothers approached did agree to participate, and this may go some way to offset this issue of representativeness. Finally, the subjective rating scale used for assessing the diagnostic experience was created for this study, and has not been used before. However, the relatively strong internal consistency scores do suggest that it may be assessing a robust construct.

The current findings have a number of implications for practice. In particular, findings of note are that the older the child at diagnosis, the worse the perceived speed, and the greater the number of professionals involved in the process, the less coherent the process was perceived by the mother. Indeed, the only significant predictor for anxiety in mothers was the number of professionals involved. This may be an important consideration for services and how they are organised. Another potentially important finding that has implications for services is that the shorter the length of the diagnostic process, the greater the levels of stress in the mothers. The implications for this may concern the levels of support or training that a mother may need in order to prepare her to be as proactive as possible during this period. These findings imply a need for increased parent resources—the other side of the coin may be that professionals may need to skill mothers in early interventions that have been demonstrated to be highly effective. 

## 5. Conclusions

Mothers of children with ASD were noted to display high levels of stress, and these levels of stress were related to worse parent health and child outcomes. Mothers found the diagnostic process particularly stressful. The current study noted that the speed and coherence reduced mothers’ anxiety but increased their parenting stress. These findings suggest that care is needed to help the parents through the diagnostic process, with regard to their own levels of functioning. 

## Figures and Tables

**Table 1:** Mean levels of autism severity (ADOS), nonverbal functioning (Leiter-R), and verbal functioning (BPVS) and their Pearson correlations.

Scale	Mean (SD; Range)	Leiter	BPVS
ADOS (CSS)	6.54 (1.81; 3–10)	−0.457 ***	−0.411 ***
Leiter-R (Standard)	70.16 (16.47; 35–109)		0.822 ***
BPVS (Standard)	63.44 (18.95; 2–104)		

*** *p* < 0.001.

**Table 2:** Spearman correlations between parent, child, and diagnostic process factors and the parents’ perceived view of the diagnostic process.

	Mean (SD)	Speed	Interpersonal Skills	Communcation	Helpfulness	Coherence	Overall
		3.63 (2.02)	4.22 (1.95)	3.61 (1.87)	3.39 (2.04)	3.84 (2.12)	18.69 (7.33)
Parent age	39.34 (8.24)	0.043	0.014	0.148	−0.115	−0.005	−0.011
Autism Spectrum Disorder (ASD) Severity	24.02 (7.13)	−0.054	−0.094	−0.066	0.101	0.036	−0.018
Intellectual Functioning	70.16 (16.47)	0.164 *	0.053	0.159 *	0.018	0.035	0.115
Verbal Functioning	63.44 (18.95)	0.208 **	0.124	0.219 **	0.087	0.132	0.209 **
Child age	8.64 (4.53)	−0.048	−0.053	−0.017	−0.109	−0.050	−0.040
Child age at diagnosis	5.45 (3.85)	−0.267 ***	−0.120	−0.100	−0.047	0.000	−0.155 *
Child age at first problem	2.41 (2.72)	−0.117	0.051	0.013	−0.008	0.085	−0.073
Time to diagnosis	35.01 (37.75)	−0.185 *	−0.110	−0.074	−0.015	0.012	−0.094
Duration of diagnosis	16.75 (11.14)	−0.148	−0.093	−0.093	0.011	0.028	−0.092
Number professionals	5.41 (2.46)	−0.081	−0.119	−0.175 *	0.100	−0.200 **	−0.156 *

* *p* < 0.05; ** *p* < 0.01; *** *p* < 0.001.

**Table 3:** Means (standard deviations for the parents self-reports of health, and the Pearson correlations between parent, child, and diagnostic factors and parents’ self-reports of health (HADS-A = anxiety; HADS-D = depression; GHQ = health relayed quality of life; QRS = parenting stress).

	HADS-A	HADS-D	GHQ	QRS
Mean	10.67	9.59	29.98	26.77
(SD)	(3.95)	(3.37)	(19.31)	(9.13)
Parent age	−0.064	0.112	−0.255 *	−0.041
Autism Severity	0.019	−0.107	0.002	0.205 *
Intellectual Functioning	−0.067	0.117	0.015	−0.318 ***
Verbal Functioning	−0.162 *	0.092	−0.063	−0.384 ***
Child age	−0.066	0.021	−0.262 **	−0.117
Child age at diagnosis	0.046	0.080	−0.104 *	−0.226 *
Child age at first problem	0.015	0.005	0.131	−0.003
Time to diagnosis	−0.003	0.053	−0.257 *	−0.308 ***
Duration of diagnosis	−0.048	0.042	−219 *	−0.306 ***
Number of professionals	0.421 ***	−0.056	0.178 *	0.068

* *p* < 0.05; ** *p* < 0.01; *** *p* < 0.001.

**Table 4:** Correlations between parent views of the diagnostic process and parents’ self-reports of health (HADS-A = anxiety; HADS-D = depression; GHQ = health relayed quality of life; QRS = parenting stress).

	HADS-A	HADS-D	GHQ	QRS
Perceived speed	−0.295 **	−0.222 **	−0.208 **	−0.064
Prof. interpersonal skill	−349 ***	−0.103	−0.275 **	−0.007
Prof communication	−0.362 ***	−0.107	−0.231 **	−0.136
Helpfulness	−472 ***	−0.227 **	−0.216 *	−0.030 *
Coherence	−0.584 ***	−0.086	−0.283 **	−0.156 **
Overall	−0.542 ***	−0.211 **	−0.294 ***	−0.155 *

* *p* < 0.05; ** *p* < 0.01; *** *p* < 0.001.
